# Enalos KNIME nodes: exploring environmental durability, ageing resistance and corrosion inhibition

**DOI:** 10.1186/1758-2946-5-S1-P42

**Published:** 2013-03-22

**Authors:** A Tsoumanis, G Melagraki, A Afantitis

**Affiliations:** 1 Department of Chemoinformatics, NovaMechanics Ltd, Nicosia, 1046, Cyprus

## 

Environmental durability, ageing and corrosion tests are applied before a product comes to the market to ensure that the product's life time behaviour is well established. Current tests demand a long period of weeks - months before safe results can be concluded based on the procedures described and implemented so far. The need for a reduction in time required for evaluation of a product's behaviour is now more than important since now there is a limited time from development until the product reaches the market. The pressure of the market, the time gap between the conception of a product and its production decreases dramatically and there is a need for better and more severe corrosion and ageing tests in order to shorten the lead-time of products. To achieve this goal, in an effort to reduce the cost and time required, within EUREKA MAAC project [1], we have computationally explored experimental data produced by our partners and from the literature. We have developed advanced mathematical models for predicting environmental durability, ageing resistance and corrosion inhibition with respect to environmental factors and material structure. For this purpose we have successfully developed KNIME workflows including Enalos KNIME nodes [2] such as Enalos Mold2 KNIME node, Enalos Model Acceptability Criteria KNIME Node and Enalos Domain KNIME Node that were implemented to calculate Mold2 descriptors, validate the produced models and define its domain of applicability respectively [3]. The proposed method, due to the high predictive ability, can be a useful aid to the design of new high-quality products with minimum investment in time and money for experimental work.
